# Supplementary material for: The Evaluation of a New ELISA-Based Kit for Total Microcystins as an Early Detection Tool for Microcystin Blooms in Source Waters and Its Application State-Wide to Oregon Source and Finished Drinking Waters
Source: Toxins (Basel). 2025 Jan 24;17(2):53. doi: 10.3390/toxins17020053 (PMC11861646; doi:10.3390/toxins17020053)
Supplement: Supplementary file 1 [file toxins-17-00053-s001.zip › toxins-3357538-supplementary/Referenced-LCMRL-MDL-Lab1.pdf]

see next page for all concentrations/levels included.

LCMRL Report for Microcystins & Nodularins ADDA/SAES

Analysis Details

Date - 05/19/2021
Contaminant Name - Microcystins & Nodularins ADDA/SAES
Analytical Method - ELISA/CAAS
Units - Micrograms/Liter (ug/L)

Data

Concentrations					
	0.015	0.02	0.03	0.05	0.075
Value 1	0.006	0.009	0.029	0.045	0.084
Value 2	0.017	0.016	0.034	0.062	0.093
Value 3	0.021	0.025	0.023		0.072
Value 4	0.017	0.013	0.03	0.05	0.061
Value 5	0.013	0.018	0.031	0.051	0.086
Value 6	0.017	0.023	0.03	0.046	0.096
Value 7	0.015	0.018	0.02	0.046	
Value 8	0.012	0.017	0.023	0.044	0.063
Value 9					0.074
Value 10					0.076
Value 11					0.075
Value 12					0.09
Value 13					0.095
Value 14					0.085
Value 15					
Value 16					
Value 17					
Value 18					

Concentrations
Value 1
Value 2
Value 3
Value 4
Value 5
Value 6
Value 7
Value 8
Value 9
Value 10
Value 11
Value 12
Value 13
Value 14
Value 15
Value 16

Concentrations

	Conc. 1	Conc. 2	Conc. 3	Conc. 4	Conc. 5	Conc. 6	Conc. 7	Conc. 8	Conc. 9	Conc. 10
	0.015	0.02	0.03	0.05	0.075	0.115	0.15	0	0.3	0.5
**Measured Concentrations**										
Value 1	0.006	0.009	0.029	0.045	0.084	0.112	0.108	0.001	0.333	0.545
Value 2	0.017	0.016	0.034	0.062	0.093	0.124	0.137	0.002	0.383	0.631
Value 3	0.021	0.025	0.023		0.072	0.146	0.233	0.007	0.348	0.509
Value 4	0.017	0.013	0.03	0.05	0.061	0.105	0.169	0.002	0.338	0.551
Value 5	0.013	0.018	0.031	0.051	0.086	0.132	0.185	0.002	0.328	0.513
Value 6	0.017	0.023	0.03	0.046	0.096	0.134	0.187	0.012	0.328	0.472
Value 7	0.015	0.018	0.02	0.046		0.096	0.120	0.002	0.318	0.494
Value 8	0.012	0.017	0.023	0.044	0.063	0.104	0.155	0.002		
Value 9					0.074	0.117		0.002		
Value 10					0.076	0.123		0.001		
Value 11					0.075	0.123		0.002		
Value 12					0.09	0.119		0.004		
Value 13					0.095	0.096		0.012		
Value 14					0.085	0.109		0.005		
Value 15								0		
Value 16										
Value 17										
Value 18										

Value 17
Value 18

Results

R:	0
R Squared:	0
Adjusted R Squared:	0.9765
	y = 0.0030654 + 0.82372 * x + 1.9309 * x^2 - 2.9479 * x^3
LCMRL:	0.21 ug/L
DL:	0.027 ug/L
Critical Level:	0.013 ug/L

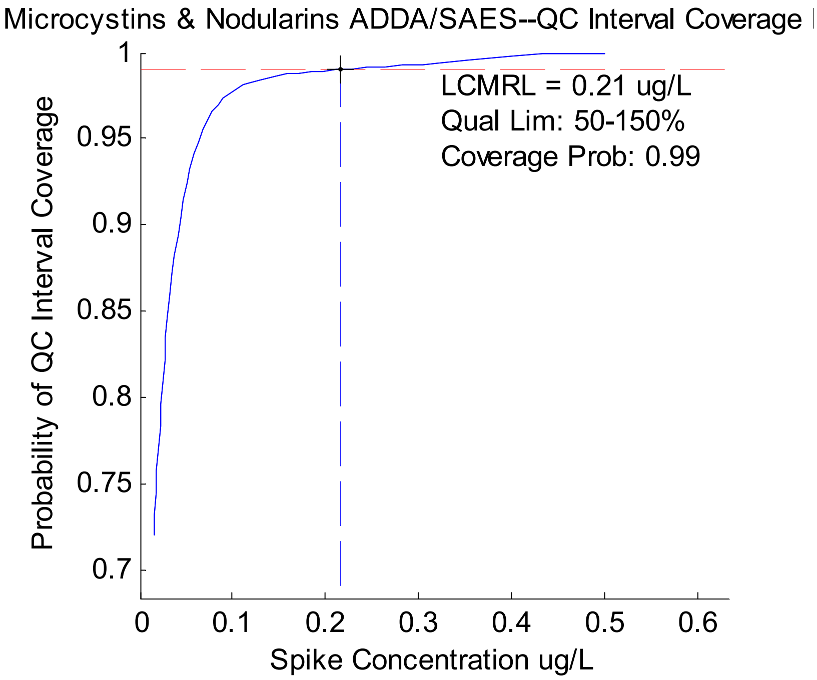
Microcystins & Nodularins ADDA/SAES--QC Interval Coverage
Probability of QC Interval Coverage
LCMRL = 0.21 ug/L
Qual Lim: 50-150%
Coverage Prob: 0.99
1
0.95
0.9
0.85
0.8
0.75
0.7
0
0.1
0.2
0.3
0.4
0.5
0.6
Spike Concentration ug/L

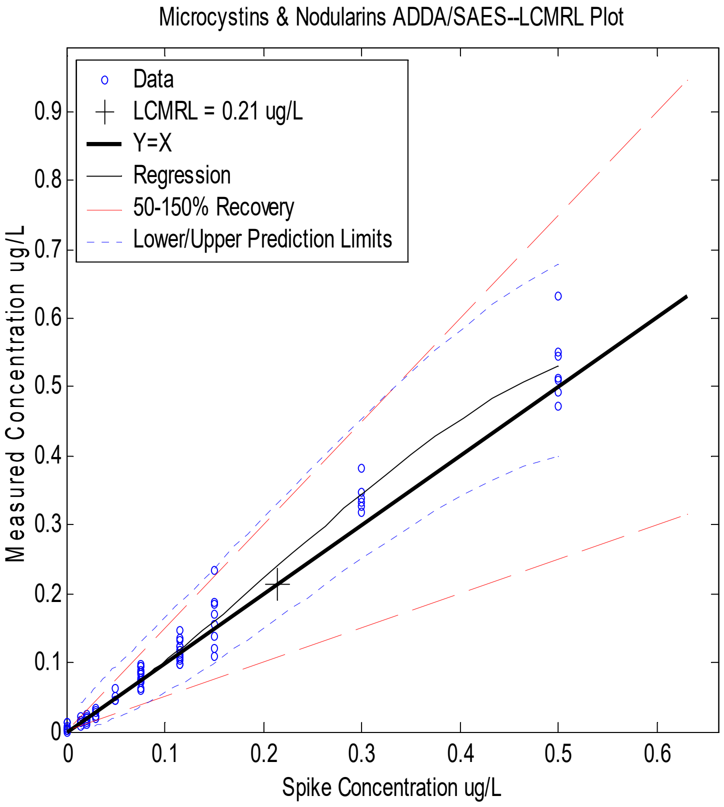
Microcystins & Nodularins ADDA/SAES--LCMRL Plot
Data
LCMRL = 0.21 ug/L
Y=X
Regression
50-150% Recovery
Lower/Upper Prediction Limits
Measured Concentration ug/L
0.9
0.8
0.7
0.6
0.5
0.4
0.3
0.2
0.1
0
0
0.1
0.2
0.3
0.4
0.5
0.6
Spike Concentration ug/L
