# Supplementary figures and images for: The Evaluation of a New ELISA-Based Kit for Total Microcystins as an Early Detection Tool for Microcystin Blooms in Source Waters and Its Application State-Wide to Oregon Source and Finished Drinking Waters
Source: Toxins (Basel). 2025 Jan 24;17(2):53. doi: 10.3390/toxins17020053 (PMC11861646; doi:10.3390/toxins17020053)

Total Microcystins (SAES) Lab OR-DEQ  
QC Interval Coverage Plot

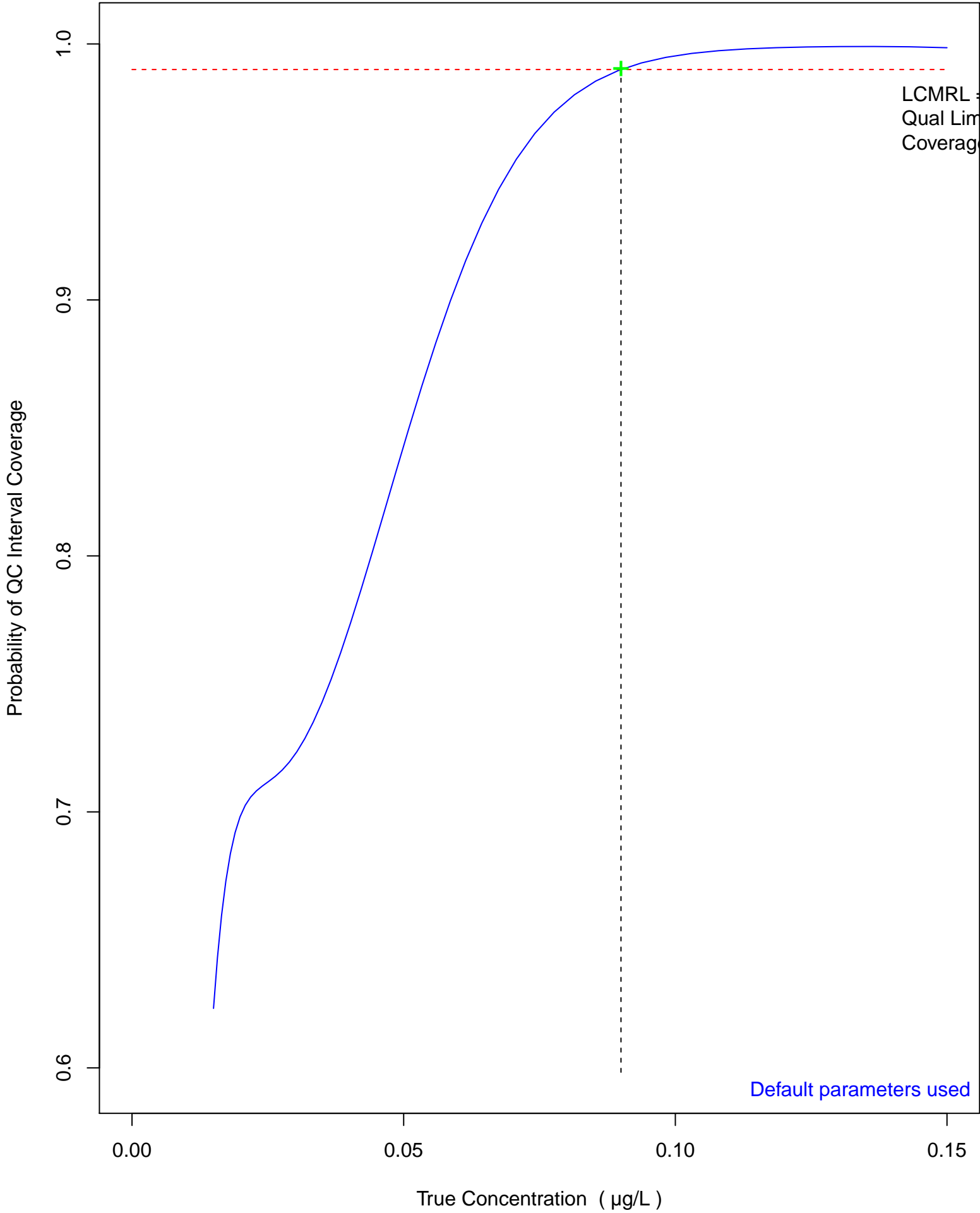

## LCMRL Plot

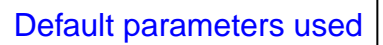

Supplement: Supplementary file 1 [file toxins-17-00053-s001.zip › toxins-3357538-supplementary/Referenced-LCMRL-MDL-Lab2.pdf]
